# Baroreflex sensitivity and outcomes following coronary surgery

**DOI:** 10.1371/journal.pone.0175008

**Published:** 2017-04-06

**Authors:** Marco Ranucci, Alberto Porta, Vlasta Bari, Valeria Pistuddi, Maria Teresa La Rovere

**Affiliations:** 1 Department of Cardiothoracic, Vascular Anesthesia and Intensive Care, IRCCS Policlinico San Donato, Milan, Italy; 2 Department of Biomedical Sciences for Health, University of Milan, Milan, Italy; 3 Department of Cardiology, IRCCS Istituti Clinici Scientifici Maugeri, Istituto di Montescano, Montescano, Pavia, Italy; Universidade de Mogi das Cruzes, BRAZIL

## Abstract

Postoperative atrial fibrillation, acute kidney dysfunction and low cardiac output following coronary surgery are associated with morbidity and mortality. The purpose of this study is to determine if the preoperative autonomic control is a determinant of these postoperative complications. This is a prospective cohort study on 150 adult patients undergoing surgical coronary revascularization with cardiopulmonary bypass. The patients received an autonomic control assessment after the induction of anesthesia. Baroreflex sensitivity was computed by spectral analysis and expressed as BRSαHF and BRSαLF for measure respectively in the high and low frequency domains. Atrial fibrillation was adjudicated at any postoperative time during the hospital stay. Acute kidney dysfunction was defined as any increase of serum creatinine levels from preoperative values within the first 48 hours after surgery, and acute kidney injury was adjudicated at a 50% increase. Low cardiac ouput syndrome was defined as the need for inotropic support > 48 hours. Thirty-eight (26.4%) patients experienced postoperative atrial fibrillation; 32 (22.2%) had acute kidney dysfunction and 5 (3.5%) acute kidney injury; 14(10%) had a low cardiac output state. No indices of baroreflex sensitivity were associated with atrial fibrillation or acute kidney injury. A low value of BRSαLF was associated with acute kidney dysfunction and low cardiac output state. A BRSαLF < 3 msec/mmHg was an independent risk factor for acute kidney dysfunction (odds ratio 3.0, 95% confidence interval 1.02–8.8, P = 0.045) and of low cardiac output state (odds ratio 17.0, 95% confidence interval 2.9–99, P = 0.002). Preoperative baroreflex sensitivity is linked to postoperative complications through a number of possible mechanisms, including an autonomic nervous system-mediated vasoconstriction, a poor response to hypotension, and an increased inflammatory reaction.

## Introduction

The arterial baroreflex is an important determinant of the neural regulation of the cardiovascular system. A reduction in the baroreceptor-heart rate reflex (i.e., baroreflex sensitivity, BRS), has been reported in hypertension, coronary artery disease, myocardial infarction and heart failure. [1] The majority of the studies have shown that lower BRS values are associated with higher cardiovascular disease-related mortality. [2–4] More specifically, it has been recently suggested that a cut-off value around 3 ms/mmHg—a threshold rather constant through different methodologies—can be viewed as a *biological threshold* for the functioning of the baroreflex. [2, 5]

A maladaptation of the autonomic nervous system (ANS) is involved in a number of post-surgical complications including atrial fibrillation (AF), acute kidney dysfunction (AKD), and injury (AKI), and low cardiac output syndrome (LCOS).

In cardiac surgery, new onset AF can be found in approximately 20% to 40% of the patient population depending on the type of surgery and the patient profile, [6,7] and it is accompanied by an increased risk of stroke and prolonged intensive care unit and hospital stay. [8] The ANS has been previously identified as an important determinant of AF [9]; however, studies analysing autonomic fluctuations preceding the onset of post-operative AF [10, 11] yielded conflicting results. [12–15]

In addition to cardiac function, the ANS is also involved in the modulation of kidney function. [16] Depending on the definitions, AKI can be found in 2%-20% of the patient population, and is invariably associated with an increased immediate and long-term mortality. [17, 18] Similarly to AF, the aetiology of renal dysfunction associated with cardiac surgery is multifactorial including operative and post-operative factors (ischemia-reperfusion injury, inflammation and oxidative stress). However, no data exist on the potential role of the autonomic control in the pathogenesis of post-operative kidney dysfunction.

Following cardiac surgery, LCOS is observed in up to 20% of the patients. [19] The inability of the ANS to activate effective circulatory reflexes to maintain hemodynamic stability is a feature of LCOS. While it is well-recognized that cardiovascular autonomic neuropathy in diabetic patients may result in unexpected hemodynamic instability during surgery, [20] very few studies have analyzed the impact of autonomic dysfunction on post/peri-operative outcomes in a general population or in cardiac surgery patients. [21, 22]

The experimental hypothesis of the present study is that the preoperative autonomic control, defined in terms of BRS, may be an independent determinant of AF, renal function impairment, and LCOS following cardiac surgery.

## Methods

Prospective cohort study performed according to the declaration of Helsinki. The study design was approved by the Local Ethics Committee (Ethics Committee San Raffaele Hospital, Milan). All the patients gave a written informed consent.

### Patients

The study population was constituted by 150 adult (> 18 years) patients undergoing elective or urgent coronary artery bypass graft (CABG) surgery with cardiopulmonary bypass (CPB). Exclusion criteria were emergency surgery, known ANS pathology, non-sinus rhythm. Withdrawal criteria were mortality within the first 48 hours from surgery and technical impossibility of recording post-anesthesia induction data.

### Anesthesia

According to our standard practice, the patients received a premedication with intramuscular atropine (0.5 mg) and fentanyl (100 μg) about 1 hour before reaching the operating theater. Anesthesia was induced with an intravenous bolus injection of propofol at 1.5 mg^.^kg^-1^ and infusion of remifentanil 0.2 μg^.^kg^-1.^min^-^1. Maintenance of anesthesia was achieved with a continuous infusion of propofol at 3 mg^.^kg^-1.^h^-1^ and a remifentanil infusion range from 0.05 to 0.5 μg^.^kg^-1.^min^-1^. Additional inhalatory agents (sevorane) could be used as requested.

### Clinical data collection and definitions

Preoperative data included demographics, co-morbidities, serum creatinine value (mg/dL), cardiovascular profile, and mortality risk stratification using the European System for Cardiac Operative Risk Evaluation (EuroSCORE II). Intraoperative data included the presence of additional surgical procedures, lowest temperature on CPB, and CPB duration. Postoperative data included in-hospital mortality, need for postoperative inotropic support, mechanical ventilation time (hours), intensive care unit (ICU) and hospital stay (days). The presence of AF was adjudicated in case of any AF event recorded during the postoperative period, from the admission to the ICU to the hospital discharge, and detected by continuous ECG monitoring during the ICU stay or by daily ECG control after discharge from the ICU.

Postoperative renal dysfunction was assessed based on the peak postoperative serum creatinine level within 48 hours from surgery. The presence of AKI was adjudicated according to the Acute Kidney Injury Network criteria as AKI stage 1 (50% increase in peak postoperative serum creatinine from baseline value or an absolute increase > 0.3 mg/dL) or higher stages, within 48 hours from surgery. AKD was defined as any increase in serum creatinine value from baseline within 48 hours from surgery. LCOS was defined as the need for inotropic support for more than 48 hours after surgery.

### Experimental data collection and definitions

The experimental protocol was already described in details. [23] Lead II ECG and arterial pressure, invasively derived from the radial artery, were acquired from patient’s monitor, through an A/D board (National Instruments, Austin, Tx) connected to a laptop. Data were recorded after the induction of general anesthesia.

The sampling frequency was 1000 Hz and the recording session lasted 10 minutes.

The beat-to-beat series of R-R interval (R-R) and systolic arterial pressure (SAP) were extracted during the entire recording period from ECG and arterial pressure respectively. R-R and SAP mean and variance were extracted and expressed in milliseconds (ms), mmHg, ms^2^ and mmHg^2^. Power spectral density was estimated through a parametric approach. [24] A spectral component was labeled as low frequency (LF) or high frequency (HF) if its central frequency was between 0.04 and 0.15 Hz or between 0.15 and 0.5 Hz, respectively.

BRS was evaluated through spectral approach. [25] BRS was computed as the square root of the ratio of the LF of R-R to the LF of SAP, or the HF to the HF of SAP, and indicated as BRSαLF and BRSαHF respectively, and expressed as ms/mmHg.

### Statistics

Data are presented as median with interquartile range for continuous, non-normally distributed variables, and as number with percentage for dichotomous variables. Normality assumption was tested using a Kolmogorov-Smirnov test. The difference between the recorded parameters in the AF and non-AF, AKD/AKI and non-AKD/AKI, and LCOS and non-LCOS groups was investigated at an univariate analysis using parametric (Student’s t test) and non-parametric (Mann Whitney U test) tests as appropriate. Differences in dichotomous variables frequencies were tested with a Fisher’s exact test or Pearson’s chi square as appropriate. The predictive ability of the identified variables was tested with a receiver operating characteristics (ROC) analysis, producing areas under the curve (AUC). Adequate cut-off values were identified according to the best coupling of sensitivity and specificity values with the pre-requisite of a specificity of t least 90%.

A subsequent multivariable analysis (logistic regression) was applied to the BRS dichotomized according to the identified cut-off value, and to all the factors having an association with the outcome variables at a P value of 0.05 or less. For each outcome, the independent risk factors were identified and expressed as odds ratios and 95% confidence interval. For all the statistical tests, a P value < 0.05 was considered statistically significant. All the statistical analyses were performed with computerized packages (SPSS 13.0, IBM, Chicago, IL, and MedCalc, MedCalc Software, Ostend, Belgium).

## Results

The general characteristics of the patients population are depicted in Tables 1 and 2. Out of the 150 patients enrolled, autonomic control parameters were computed in 144 patients who constituted the study population. In the remaining 6 patients it was not possible to extract the variability indices due to frequent arrhythmias or bad arterial pressure recording. Thirty-eight (26.4%) patients experienced at least one episode of postoperative AF, and 14 (9.7%) a LCOS, whereas 32 (22.2%) fulfilled the criteria for AKD and 7 (4.8%) those for AKI. Patients with AKD had a significantly lower hematocrit and higher EuroSCORE II; they experienced a longer mechanical ventilation time and ICU stay; patients with AKI had the same profile plus a significantly smaller weight.

**Table 1:** Patient characteristics, surgical details, and outcome in the overall patient population (N = 144) and according to the presence of AKD and AKI.

Variables	Value (overall)	AKD (N = 32)	No AKD (N = 112)	P	AKI (N = 7)	No AKI (N = 137)	P
Age (years)	67 (59–74)	70 (63–76)	66 (58–73)	0.080	72 (63–88)	67(59–74)	0.111
Gender female	19 (13)	6 (19)	13 (11.6)	0.292	2 (29)	17 (12)	0.231
Weight (kgs)	77 (68–86)	74 (68–87)	78 (69–86)	0.432	70 (65–70)	78 (69–87)	0.011
Congestive heart failure	5 (3.5)	2 (6.3)	3 (2.7)	0.330	0 (0)	5 (3.6)	0.607
Recent myocardial infarction	19 (13.2)	4 (12.5)	15 (13.4)	0.893	0 (0)	19 (14)	0.594
Ejection fraction (%)	54 (48–60)	51 (46–67)	55 (49–60)	0.304	50 (50–60)	55 (48–60)	0.929
Diabetes	44 (30.6)	12 (37.5)	32 (28.6)	0.334	4 (57)	40 (29)	0.201
COPD	11 (7.6)	3 (9.4)	8 (7.1)	0.675	0 (0)	11 (8)	0.435
Serum creatinine (mg/dL)	1.0 (0.9–1.1)	1.0 (0.8–1.2)	1 (0.9–1.1)	0.684	0.8 (0.3–1.5)	1 (0.9–1.1)	0.476
Hypertension	88 (61.1)	22 (68.8)	56 (58.9)	0.313	5 (71)	83 (61)	0.706
Previous cerebrovascular accident	9 (6.3)	2 (6.3)	7 (6.3)	1.000	1 (14)	8 (5.8)	0.370
HCT (%)	38.8 (36–42)	36 (33–39)	40 (37–43)	0.001	36 (35–37)	39 (36–42)	0.016
ACE inhibitors	44 (30.6)	12 (37.5)	32 (28.8)	0.349	4 (57)	40 (29)	0.202
Beta-blockers	83 (57.6)	20 (62.5)	63 (56.3)	0.528	4 (57)	79 (58)	1.000
Calcium antagonists	8 (5.6)	0 (0)	8 (7.1)	0.120	0 (0)	8 (5.8)	0.511
Amiodarone	11 (7.6)	2 (6.5)	9 (8.0)	0.770	0 (0)	11 (8.1)	0.424
Associated mitral valve repair	3 (2.1)	0 (0)	3 (2.7)	0.349	0 (0)	3 (2.2)	0.692
EuroSCORE II	1.3 (1–2.3)	1.9 (1.2–3.1)	1.2 (0.9–1.8)	0.001	2.3 (1.9–3.8)	1.2 (1–2.1)	0.008
CPB time (min)	58 (49–76)	58 (46–76)	58 (51–76)	0.904	56 (47–61)	58 (49–76)	0.382
Nadir temperature (°C) on CPB	33 (32–33.4)	33 (32–33.7)	33 (32–33.4)	0.868	33 (32–33)	33 (32–33)	0.444
Mechanical ventilation time (h)	12 (8–16)	14 (11–18)	11 (8–16)	0.017	18 (14–40)	11 (8–16)	0.003
Intensive care unit stay (d)	1 (1–3)	2.5 (1–4)	1 (1–3)	0.020	3 (1–5)	1 (1–3)	0.455
Hospital stay (d)	7 (6–8)	7 (6–9)	7 (6–9)	0.590	7 (4–8)	7 (6–9)	0.182
30-days mortality	2 (1.4)	1 (3.1)	1 (0.9)	0.341	1 (14.3)	1 (0.7)	0.095

Continuous data are presented as median (interquartile range); categorical data as number (%). ACE: angiotensin converting enzyme;

AKD: acute kidney dysfunction; AKI: acute kidney injury; CPB: cardiopulmonary bypass; COPD: chronic obstructive pulmonary disease; HCT: hematocrit.

**Table 2:** Patient characteristics, surgical details, and outcome in the overall patient population (N = 144) and according to the presence of LCOS and AF.

Variables	Value (overall)	LCOS (N = 14)	No LCOS (N = 130)	P	AF (N = 38)	No AF (N = 106)	P
Age (years)	67 (59–74)	65 (59–76)	67 (59–74)	0.840	73 (64–77)	66 (58–71)	0.001
Gender female	19 (13)	2 (14)	17 (13)	0.899	10 (26.3)	9 (8.5)	0.005
Weight (kgs)	77 (68–86)	78 (69–86)	77 (68–86)	0.981	75 (68–81)	78 (68–88)	0.147
Congestive heart failure	5 (3.5)	0 (0)	5 (3.8)	0.455	2 (5.3)	3 (2.8)	0.608
Recent myocardial infarction	19 (13.2)	1 (7.1)	18 (14)	0.694	5 (13.2)	14 (13.2)	1.000
Ejection fraction (%)	54 (48–60)	44 (31–50)	55 (50–60)	0.001	54 (50–60)	54 (50–60)	0.666
Diabetes	44 (30.6)	6 (43)	38 (29)	0.293	10 (26.3)	34 (32.1)	0.508
COPD	11 (7.6)	3 (21)	8 (6.2)	0.076	2 (5.3)	9 (8.5)	0.728
Serum creatinine (mg/dL)	1.0 (0.9–1.1)	1.1 (0.9–1.4)	1 (0.9–1.1)	0.044	1 (0.9–1.1)	1 (0.9–1.1)	0.858
Hypertension	88 (61.1)	9 (74)	69 (71)	0.798	24 (63.2)	64 (60.2)	0.763
Previous cerebrovascular accident	9 (6.3)	1 (7.1)	8 (6.2)	0.885	4 (10.5)	5 (4.7)	0.244
HCT (%)	38.8 (36–42)	40 (36–44)	39 (36–42)	0.539	37 (35–39)	40 (37–43)	0.001
ACE inhibitors	44 (30.6)	6 (43)	38 (29)	0.302	14 (36.8)	30 (28.6)	0.344
Beta-blockers	83 (57.6)	8 (57)	75 (58)	1.000	23 (60.5)	60 (56.6)	0.675
Calcium antagonists	8 (5.6)	1 (0.7)	7 (4.9)	0.568	1 (2.6)	7 (6.6)	0.359
Amiodarone	11 (7.6)	1 (7.1)	10 (7.8)	1.000	3 (8.1)	8 (7.5)	0.912
Associated mitral valve repair	3 (2.1)	2 (14)	1 (0.8)	0.025	1 (2.6)	2 (1.9)	1.000
EuroSCORE II	1.3 (1–2.3)	3.1 (1.3–3.6)	1.2 (1–2)	0.002	1.5 (1–2.6)	1.2 (1–2.1)	0.074
CPB time (min)	58 (49–76)	61 (51–78)	57 (49–76)	0.424	57 (48–73)	58 (50–76)	0.698
Nadir temperature (°C) on CPB	33 (32–33.4)	32 (32–33)	33 (32–33)	0.382	32 (32–32.2)	33 (32–33.5)	0.730
Mechanical ventilation time (h)	12 (8–16)	17 (14–21)	11 (8–14)	0.001	11.5 (9–17.2)	12 (8–16)	0.384
Intensive care unit stay (d)	1 (1–3)	4 (3–5)	1 (1–3)	0.001	2 (1–4)	1 (1–3)	0.023
Hospital stay (d)	7 (6–8)	9 (8–11)	7 (6–8)	0.001	8 (7–10)	7 (6–8)	0.005
30-days mortality	2 (1.4)	1 (7.1)	1 (0.8)	0.186	1 (2.6)	1 (0.9)	1.000

Continuous data are presented as median (interquartile range); categorical data as number (%). ACE: angiotensin converting enzyme;

AF: atrial fibrillation; CPB: cardiopulmonary bypass; COPD: chronic obstructive pulmonary disease; HCT: hematocrit; LCOS: low cardiac output state.

Patients with LCOS had a significantly lower ejection fraction, higher serum creatinine values and EuroSCORE II, and were significantly more likely to receive an associated mitral valve repair. They had a significantly longer mechanical ventilation time, ICU stay, and hospital stay. Patients with AF were significantly older, more often females and with a lower hematocrit, and had a significantly longer ICU and hospital stay.

Table 3 reports the BRS data in patients with or without AKD, AKI, LCOS and AF. Overall, BRS in the HF domain was not significantly different in patients with or without any of the considered bad outcomes. Conversely, patients who experienced an AKD had significantly (P = 0.006) lower levels of BRS in the low frequency domain (αLF) and the same applies (P = 0.029) to patients who experienced an LCOS.

**Table 3:** BRS in the overall patient population (N = 144) and according to the presence of cardiac complications.

Patient population	αHF (ms/mmHg)	P	αLF (ms/mmHg)	P
Overall	4.5 (2.6–8.0)		8.3 (4.6–15.1)	
Atrial fibrillation				
Yes (N = 38)	5.0 (3.5–10.4)		9.8 (4.0–17.8)	
		0.184		0.594
No (N = 106)	4.0 (2.5–7.3)		7.8 (4.6–14.5)	
Acute kidney dysfunction				
Yes (N = 32)	3.8 (2.1–8.9)		6.0 (3.1–9.9)	
		0.364		**0.006**
No (N = 112)	4.6 (2.9–7.7)		9.1 (5.6–18.1)	
Acute kidney injury				
Yes (N = 7)	2.6 (0.97–4.8)		3.9 (1.8–22.4)	
		0.123		0.137
No (N = 137)	4.6 (2.6–8.1)		8.4 (4.7–15.1)	
Low cardiac output state				
Yes (N = 14)	2.6 (1.4–6.9)		4.1 (0.89–9.8)	
		0.054		**0.029**
No (N = 130)	4.6 (2.7–8.1)		8.4 (4.8–16.1)	

Data are presented as median (interquartile range). BRS: baroreflex sensitivity; αHF: BRS in the high frequency band; αLF: BRS in the low frequency band

**Fig 1:**
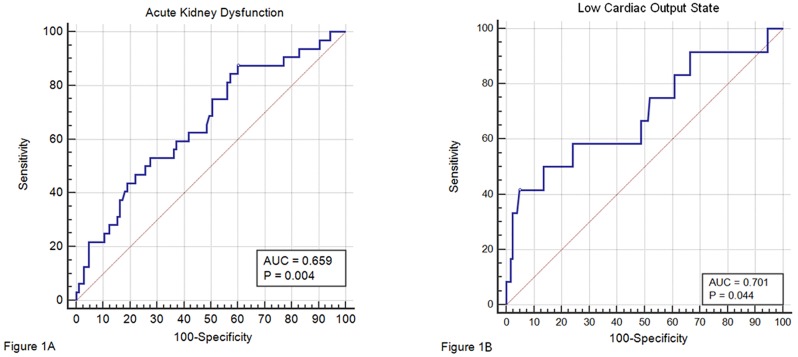
Receiver operating characteristics curve of BRSαLF as predictor of acute kidney dysfunction and low cardiac output state. Receiver operating characteristics curve of BRSαLF (baroreflex sensitivity low frequency) as predictor of acute kidney dysfunction (panel A) and low cardiac output state (panel B). AUC: area under the curve.

The predictive properties of BRSαLF for AKD and LCOS were investigated with an ROC analysis (Fig 1). The discriminatory power was moderate for both the outcomes (AUC 0.66 and 0.70 respectively). The best cut-off value for BRSαLF as predictor of AKD was identified at 2.83 ms/mmHg and as predictor of LCOS 2.65 ms/mmHg. Therefore, the αLF was dichotomized at a level < 3.0 ms/mmHg given the correspondence with a well-validated value. [5] Twenty patients had a BRSαLF < 3.0 ms/mmHg. These patients were significantly (P = 0.002) older than those with a BRSαLF ≥ 3.0 ms/mmHg (72.4±9.5 years vs. 65.2±9.5 years). No other significant differences were noticed.

The multivariable predictive models for AKD and LCOS are reported in Table 4. After correction for the other confounders (those significantly associated with the outcomes plus age as an adjustment factor), a BRSαLF < 3.0 ms/mmHg remained an independent predictor of AKD (odds ratio 3.0, 95% confidence interval 1.02–8.8, P = 0.045) and LCOS (odds ratio 17, 95% confidence interval 2.9–99, P = 0.002).

**Table 4:** Multivariable models (logistic regression analysis) for acute kidney dysfunction and low cardiac output state.

Acute kidney dysfunction			
Factor	Regression coefficient	Odds ratio (95% C.I.)	P
Preoperative hematocrit (%)	-0.220	0.80 (0.72–0.90)	0.001
Age (years)	-0.002	1.00 (0.95–1.05)	0.924
EuroSCORE II	0.063	1.1 (0.84–1.34)	0.599
BRSαLF < 3.0 ms/mmHg	1.098	3.0 (1.02–8.8)	0.045
Low cardiac output state			
Factor	Regression coefficient	Odds ratio (95% C.I.)	P
Age (years)	-0.013	0.99 (0.91–1.07)	0.747
Left ventricular ejection fraction (%)	-0.119	0.89 (0.83–0.85)	0.001
Serum creatinine (mg/dL)	-0.069	0.93 (0.26–3.4)	0.916
CPB duration (min)	0.015	1.01 (0.97–1.05)	0.306
EuroSCORE II	0.130	1.14 (0.82–1.6)	0.434
BRSαLF < 3.0 ms/mmHg	2.836	17.0 (2.9–99)	0.002

BRSαLF: baroreflex sensitivity in the low frequency band; CPB: cardiopulmonary bypass: C.I.: confidence interval.

## Discussion

The main results of our study are: (i) BRS as determined by the αHF is not associated with any of the considered outcomes, (ii) conversely, the BRSαLF is an independent predictor of AKD and LCOS, and (iii) postoperative AF is not associated with preoperative measures of BRS.

Even if negative with respect to AF, our study offers new insights into the role of baroreflex dysfunction in the development of clinical complications following cardiac surgery. Impaired baroreflex function results in increased sympathetic activity and vagal withdrawal. Several mechanisms can account for the detrimental effects of a depressed BRS (in the context of cardiac surgery). Besides the well-known electrophysiological effects of vagal and sympathetic activity, [26] baroreflex mediated increase in sympathetic activity and/or reduced vagal activity may contribute to increased end-organ damage and to the progression of the underlying disease. Experimental studies in animal models of BRS dysfunction induced by sino-aortic denervation provide clues into the functional and molecular consequences of autonomic dysregulation. In one study, in rats with a previous myocardial infarction, baroreceptor denervation was associated with a worse cardiac remodeling and increased mortality. [27] In a similar model but with intact hearts, baroreceptor denervation resulted in diastolic dysfunction associated with a reduction of the expression of the regulatory proteins involved in Ca^2+^ homeostasis. [28] Ackland and associates [29] analyzed molecular mechanisms linking autonomic dysfunction with poor clinical outcomes in the post-operative state in rats and found that baroreceptor denervation was associated with increased cardiac oxidative stress and impaired inotropic response through G-protein-coupled receptor kinase 2 (GRK2) upregulation. [29] The same Authors provided the clinical counterpart of this observation in a group of patients undergoing major surgery. [29] They found that the presence of parasympathetic dysfunction was associated with both a higher rate of post-operative complications and increased GRK2 expression in circulating mononuclear cells obtained preoperatively.

The exquisite sensitivity of baroreceptors to changes in arterial pressure implies that baroreflex mechanisms come into play any time a concurrent pathological event results in a transient decline in blood pressure. The sequence of events initiated by hypotension leads to a vagal withdrawal and to a generalized increase in sympathetic activity that favours a return of arterial pressure toward normal. On this background, inadequate baroreflex-mediated sympatho-excitation during a rapid rhythm [30] or an infectious disease [31] may be the leading cause of an unfavourable hemodynamic profile. Actually, in patients with post-infarction sustained ventricular tachycardia (VT), those patients presenting with syncope or signs of shock during the VT had a significantly lower BRS than patients who tolerated the arrhythmia. Only BRS, but not age or left ventricular function was related to the hemodynamic tolerability of the VT. [32] Moreover, a depressed BRS was also found as an independent predictor of mortality in post-myocardial infarction patients with preserved left ventricular function. [33]

All the above mentioned mechanisms may account for the independent association between BRS and LCOS found in our study. Of notice, BRSαLF remains independently associated with LCOS even after correction for the preoperative factor (left ventricular ejection fraction) generally considered the main predictor of LCOS.

The second finding of our study is the independent association of BRSαLF with AKD. Although the limited number of events (seven) did not allow to find associations between the BRSαLF and the more clinically relevant pattern of AKI stage 1, the finding of an independent association between BRS and minor degrees of renal dysfunction is not deprived of clinical relevance. As a matter of fact, even a minimal increase of serum creatinine levels after cardiac surgery is a determinant of increased early and long-term mortality. [34] AKD in patients with a low BRS may be a consequence of a low cardiac output state and/or of a kidney vasculature vasoconstriction and reduced renal blood flow. However, other interpretations are possible. Increasing evidence highlights the role of the vagus nerve in the regulation of immune function and inflammation through the "cholinergic anti-inflammatory pathway". [35] Among the several factors implicated in the pathogenesis of cardiac surgery-associated renal dysfunction, release of inflammatory mediators plays a significant role. A recent study demonstrated a protective effect of vagus nerve stimulation with activation of the cholinergic anti-inflammatory pathway in the ischemia reperfusion model of acute kidney injury. [36]

Our data support a recent study in high-risk surgical patients showing that baroreflex dysfunction might contribute to the development of post-operative morbidity. [22] The cut-off value for a depressed BRS in this study was set at < 6 ms/mmHg, higher than the one of the present study, and the cut-off for severely depressed BRS was set at < 3 ms/mmHg, like in our series. The differences in BRS measuring techniques (the sequence method technique in the study of Toner and associates [22], and the spectral method in ours) can account for this difference. Moreover, all our patients undergoing cardiac surgery had coronary artery disease. Although only a limited proportion had a previous myocardial infarction or heart failure, a depressed BRS has been documented also in isolated even asymptomatic coronary artery disease [37, 38].

### Limitations and clinical implications

There are some limitations in our study. The patient population was at low risk and many co-morbidities were probably underestimated being consequently a possible source of undetected bias. The low rate of AKI events did not allow us for a comprehensive statistical analysis. Finally, the reader must consider that the measurements done were certainly affected by the effects of anesthesia drugs, and namely by propofol, which is known to depress BRS. [23] For future studies, it would be highly informative to obtain autonomic nervous system parameters in the conscious patient days before surgery.

Although the present study, lacking a prospective validation, should be mainly regarded as a “proof of concept” study, we can draw some clinical considerations. By anticipating the assessment of BRS at an earlier stage in the evaluation of candidates to cardiac surgery, this would allow to take advantage of a number of measures that can improve the autonomic balance. Exercise training is known to improve BRS [39] even in the elderly, [40] and experience on the effects of a pre-operative exercise-based rehabilitation program are currently ongoing. [41] While exercise training might not be practicable on an extensive basis in the cardiac surgery population, the emerging opportunity of non-invasively modulating the parasympathetic outflow to the heart by a transcutaneous device ay the auricular level deserves to be assessed in next future. [36, 42]

## Supporting information

S1 File BRS original data set. Original data set including all variables for the overall population (N = 144).
(SAV)
