# Correction: Baroreflex sensitivity and outcomes following coronary surgery

**DOI:** 10.1371/journal.pone.0193038

**Published:** 2018-02-12

**Authors:** Marco Ranucci, Alberto Porta, Vlasta Bari, Valeria Pistuddi, Maria Teresa La Rovere

There are errors in the Funding section. The correct funding information is as follows: This study was supported by an Italian Ministry of Health Grant GR-2013-02356272 to V. Bari.
